# Measured distance from posterior tibia to popliteal artery increases with flexion and subluxation of the knee

**DOI:** 10.1002/jeo2.12070

**Published:** 2024-07-02

**Authors:** Marc Hungerford, Carmen P. Pichard‐encina, Ashlie Boner, Lynne Jones

**Affiliations:** ^1^ Orthopedics and Joint Replacement Mercy Medical Center Baltimore Maryland USA; ^2^ Johns Hopkins University School of Medicine Baltimore Maryland USA; ^3^ Medstar Good Samaritan Hospital Baltimore Maryland USA; ^4^ Orthopaedic Surgery, Johns Hopkins University School of Medicine Johns Hopkins Bayview Medical Center Baltimore Maryland USA

**Keywords:** knee flexion, knee subluxation, popliteal artery injury

## Abstract

**Purpose:**

Popliteal artery laceration is a devastating complication in total knee arthroplasty (TKA). Its anatomic position relative to the tibia has been studied using ultrasound or magnetic resonance imaging. This is the first study performed in a laboratory using radiographic measurements to determine if increased flexion and subluxation of the knee increase the distance between the tibia and popliteal artery.

**Methods:**

The femoral artery was infused with radiopaque dye in six cadavers. The knee was placed in two different degrees of flexion and three of subluxation. The radiographic distance between standardized markers in the posterior tibia and popliteal artery was measured.

**Results:**

The average distance from the tibial peg to the popliteal artery at 90° of flexion increased from 0% to 50% to 100% subluxation. The increase was statistically significant (Friedman test *p* = 0.016). The contrast between neutral and 100% subluxation was statistically significant (Sign test *p* = 0.031). At 115° flexion, average distance from the peg to popliteal artery significantly increased as subluxation increased (Friedman test *p* = 0.05). In three specimens, at 115° of flexion and 100% subluxation, a line perpendicular to the axis of the tibia, failed to intersect the popliteal artery. The measured distance increased from 90° to 115° of flexion at a given degree of subluxation, but this difference did not reach statistical significance.

**Conclusions:**

Increasing flexion and subluxation of the tibia results in increasing distance between the cut plane of the tibial plateau and popliteal artery and decreases risk of laceration.

**Level of Evidence:**

Not applicable.

**Abbreviations**
PCL: posterior cruciate ligament
TKA: total knee arthroplasty

## BACKGROUND

Injury to the popliteal artery during knee arthroplasty is a rare complication. The incidence of this complication has been reported at 0.03%–0.06% [18]. The consequences of a popliteal artery laceration can be devastating, frequently requiring revascularization surgery and even amputation.

The popliteal artery arises from the deep femoral artery as it passes through the adductor hiatus at the posterior‐superior‐medial aspect of the popliteal fossa. It then descends through the fossa obliquely until it bifurcates into the anterior and posterior tibial arteries at the posterior‐inferior‐lateral aspect of the fossa. At the level of the joint line, several studies have shown the artery to be either midline or slightly lateral to the midline, near the posterior horn of the lateral meniscus [7, 13, 17, 26]. In the popliteal fossa, the artery is fairly mobile, surrounded by fat and loose connective tissue. It is tethered proximally at the adductor hiatus and distally at the bifurcation. Vernon et al [25] studied the movement of the popliteal artery with knee flexion in cadavers. They reported that the backward shift in the artery was made possible by the conformation of the middle geniculate artery. This artery is coiled in extension and uncoils in flexion allowing the popliteal artery to move posteriorly.

In traditional knee replacement, the tibia is cut with the knee flexed and the tibia subluxed anteriorly. This was thought to protect the popliteal artery from laceration during tibial cutting by allowing the artery to fall away posteriorly. With the advent of less invasive techniques, surgeons may not flex the knee or subluxate the tibia as much, prior to cutting. Some techniques, such as the quad‐sparing technique, require cutting of the tibia first, through a small operative window using minimal knee flexion [15].

Therefore, because of the varied newer approaches utilized for total knee arthroplasty (TKA), described above, we performed this cadaver‐based study for the purpose of examining the relationship of the popliteal artery to the posterior cortex of the tibia during proximal tibial resection in various degrees of flexion and subluxation of the tibia.

## METHODS

We utilized six fresh frozen cadaver lower limbs mounted in a hinged apparatus with a rod cemented into the femur and the foot of the specimen mounted in a movable leg holder (Stulberg leg positioner, Innomed, Savannah and Georgia). The apparatus allowed the limb to be moved into a given degree of knee flexion and locked.

A median parapatellar approach to the knee was performed. The patella and extensor mechanism were subluxed laterally, the medial and lateral menisci were sharply resected, as was the anterior cruciate ligament.

A sharp retractor was placed at the lateral plateau, and a second retractor was placed anterior to the femur and posterior to the tibial plateau just lateral to the posterior cruciate ligament (PCL) for complete visualization of the anterior part of the knee. To identify the centre of the posterior edge of the tibial plateau, a headed pin was driven into the tibia at the medial aspect of the tibial insertion of the PCL.

The femoral artery was instrumented with a balloon catheter (Boston Scientific). The knees were held at either 90° or 115° of flexion, as measured with a hand‐held goniometer and either 0%, 50% or 100% of tibiofemoral subluxation. With 100% subluxation, the tibial plateau was fully dislocated in front of the medial femoral condyle. With 50% subluxation, the tibia was partially subluxed and estimated 50% between reduced and dislocated positions. Care was taken to minimize external rotation (goal 0°) with subluxation of the tibia. A size calibration marker was placed on the lateral femoral condyle. The femoral artery was infused with 10 mL of radiocontrast dye (Omnipaque, GE Healthcare) and a single lateral radiograph was obtained.

**Figure 1:**
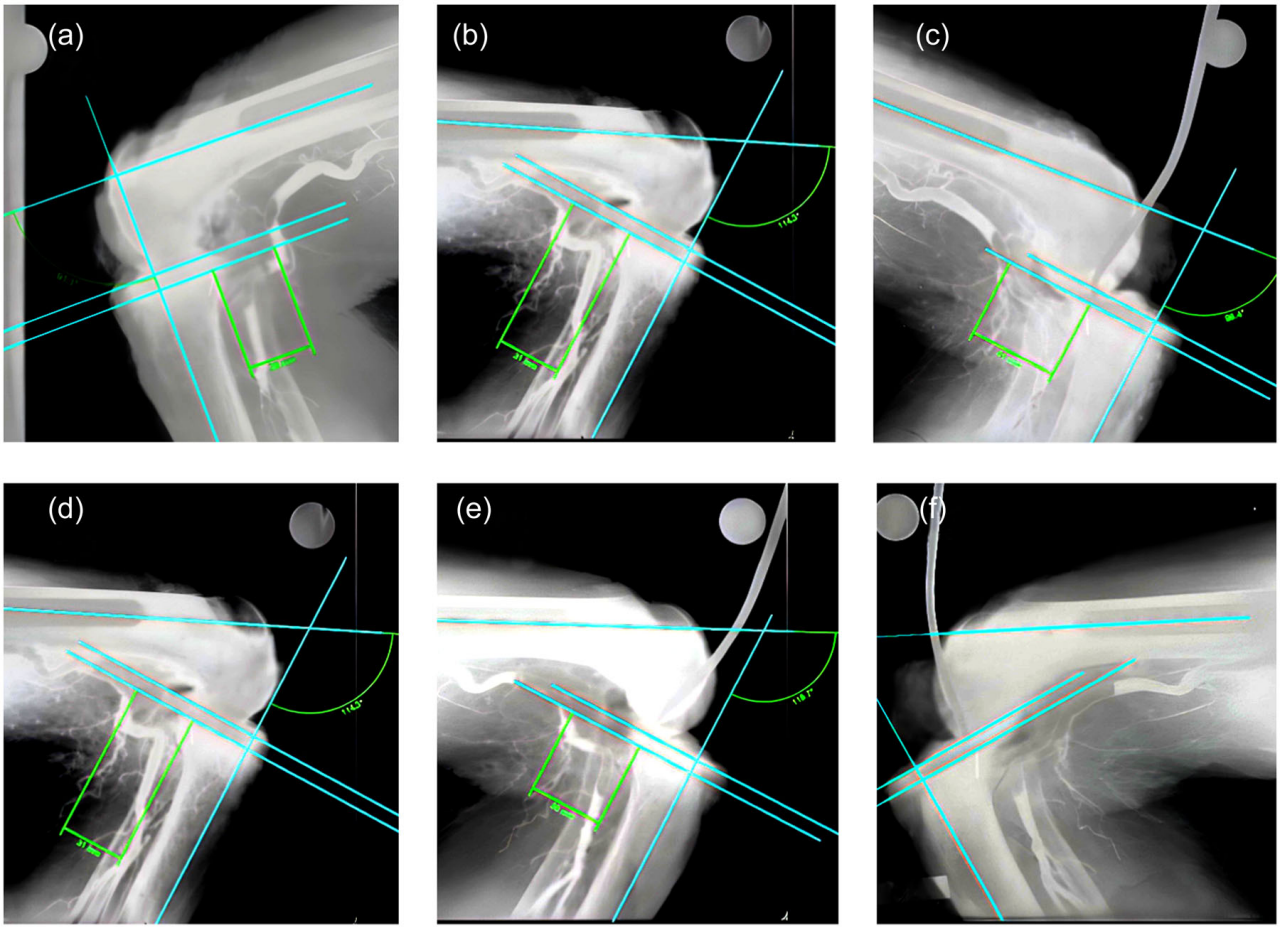
Radiographic measurements showing the distance from the point the resection plane crossed the posterior cortex of the tibia to the point it intersected the popliteal artery. 90° of flexion and no subluxation (a); 90° of flexion and 50% subluxed (b); 90° of flexion 100% subluxed (c); 115° flexion and no subluxation (d); 115° flexion and 50% subluxed (e); 115° flexion and 100% subluxed (f). This specimen shows a non‐intersection. (a) X‐ray measurements of representative specimens measuring the distance from the point the resection plane crossed the posterior cortex of the tibia to the point it intersected the popliteal artery at 90° of flexion and no subluxation. (b) X‐ray measurements of representative specimens measuring the distance from the point the resection plane crossed the posterior cortex of the tibia to the point it intersected the popliteal artery at 90° of flexion and 50% subluxed. (c) X‐ray measurements of representative specimens measuring the distance from the point the resection plane crossed the posterior cortex of the tibia to the point it intersected the popliteal artery at 90° of flexion 100% subluxed. (d) X‐ray measurements of representative specimens measuring the distance from the point the resection plane crossed the posterior cortex of the tibia to the point it intersected the popliteal artery at 115° flexion and no subluxation. (e) X‐ray measurements of representative specimens measuring the distance from the point the resection plane crossed the posterior cortex of the tibia to the point it intersected the popliteal artery at 115° flexion and 50% subluxed. (f) X‐ray measurements of representative specimens measuring the distance from the point the resection plane crossed the posterior cortex of the tibia to the point it intersected the popliteal artery at 115° flexion and 100% subluxed. This specimen showed non‐intersection.

Radiographs were digitized using the Vidar DiagnosticPro Advantage digitizer (Vidar Systems) and software. Radiographs were analysed using Endomap templating software (Siemens AG, Medical Solutions). Each image was calibrated for size using an embedded size calibration marker. The true flexion angle was measured at the intersection between a line through the axis of the femur and the axis of the tibia. To simulate the cut plane of the tibia with a 7° slope and a 7‐mm resection depth, a line was drawn at an 83° angle to the tibial shaft and through the head of the headed pin. A second line was drawn parallel to this one, but 7 mm further distally. Finally, the distance from the point this resection plane crossed the posterior cortex of the tibia to the point where it intersected the popliteal artery was measured and recorded (Figure 1). A series of measurements were carried out by two independent investigators (Table 1). Average measurements and standard deviation are given in Table 1. Statistical analyses were accomplished using STATA 12 (StataCorp). Data were analysed for descriptive measures with mean and median distances (mm) reported. The Friedman test was used to determine whether there were significant differences between median values of distance for the three percentages of subluxation for a given degree of knee flexion. A measurement of 100 mm was used as a substitute for the infinity measure. To determine the presence of differences between pairs of median intersection measurements at a given degree of knee flexion (0%–50% subluxation, 50%–100% subluxation and 0%–100% subluxation), a two‐group non‐parametric Sign test was used. To determine the presence of differences between median intersection measurements at a given percentage of subluxation at 90° and 115° of knee flexion, a two‐group non‐parametric sign test was used. The use of the sign test negates the magnitude of the upper limit of measurement and tests only whether they are greater or less than the corresponding pair. The hypotheses considered two‐tailed tests and *p* values reported. Statistical significance was set at *p* ≤ 0.05. To assess the consistency of measurements between observers, intraclass coefficients were calculated. Cohen's d calculations for sample size to provide an 80% chance of detecting a significant difference (*p* < 0.05) between groups given expected effect sizes 0.5, 1.0 and 1.5 were performed.

**Table 1:** Series of measurements carried out by two independent investigators and statistical results.

	0			50			100		
	Observer 1	Observer 2	Average	Observer 1	Observer 2	Average	Observer 1	Observer 2	Average
90° Flexion									
Specimen 1	31	28	**29.5**	39	29	**34**	32	40	**36**
Specimen 2	33	27	**30**	34	31	**32.5**	41	36	**38.5**
Specimen 3	16	18	**17**	16	17	**16.5**	29	28	**28.5**
Specimen 4	18	21	**19.5**	15	19	**17**	20	24	**22**
Specimen 5	13	13	**13**	16	15	**15.5**	37	23	**30**
Specimen 6	18	20	**19**	25	22	**23.5**	34	32	**33**
Total (mean, SD [median])			21 ± 7 [19.25]			23 ± 8 [20.25]			31 ± 6 [31.5]*
115° Flexion									
Specimen 1	23	31	**27**	27	27	**27**	38	35	**36.5**
Specimen 2	34	34	**34**	45	45	**45**	55	∞	∞
Specimen 3	21	19	**20**	21	21	**21**	36	31	**33.5**
Specimen 4	23	25	**24**	30	30	**30**	34	36	**35**
Specimen 5	25	29	**27**	38	38	**38**	∞	∞	∞
Specimen 6	50	48	**49**	∞	∞	**∞**	∞	∞	∞
Total (mean, SD [median])			30 ± 10 [27]			Undetermined [34]			Undetermined [68.25]

*Note*: Boldface represents the average values.

* *p* Value for sign test for 0–100 contrast (med. 19.25 vs. 31.5) = 0.031.

## RESULTS

Measurements are given in Table 1 and statistical results are given in Tables 2, 3, 4. Cohen's *d* calculations for sample sizes for expected size effects of 0.5, 1.0 and 1.5 were 63, 16 and 7, respectively.

**Table 2:** Comparison of values of flexion within 90° and 115° of subluxation.

Subluxation	90°	115°
Friedman test	**0.016**	**0.05**
Sign test		
0–50	0.219	0.375
0–100	**0.031**	0.063
50–100	0.219	0.625

*Note*: Bold values indicate statistically significant results.

**Table 3:** Comparison of values of flexion within degrees of subluxation.

Sign test	
	90–115° Flexion
0%	0.219
50%	0.063
100%	0.219

**Table 4:** Intraclass correlation coefficients between two observers for each of the six groups.

90	0	0.936	
	50	0.919	
	100	0.581	Infinity data
115	0	0.965	
	50	1.000	
	100	−0.444	infinity data

The average distance from the tibial peg to the popliteal artery at 7 mm below the joint line at 90° of flexion was 21.0 ± 7.0 mm at neutral, 23.0 ± 8.0 mm at 50% subluxation, and 31.0 ± 6.0 mm at 100% subluxation (mean ± SD) (Table 1). At 90° of flexion, there was a statistically significant difference in median distance measured from the peg to the popliteal artery with increasing subluxation (Friedman test *p* = 0.016). The contrast between neutral and 100% subluxation was statistically significant (med. 19.25 vs. 31.5; Sign test *p* = 0.031).

At 115° flexion, average distance from the peg to the popliteal artery increased, with increased subluxation. In three specimens, at 115° of flexion and 100% subluxation, a line perpendicular to the axis of the tibia, 7 mm below the joint line, failed to intersect the popliteal artery. One knee did not intersect at either the 50% or 100% subluxation level. At 115° of flexion, there was a statistically significant difference in median distance measured from the peg to the popliteal artery with increasing subluxation (Friedman test *p* = 0.05). There was no statistically significant difference with subluxation for the knees at 115° of flexion, using two‐group, non‐parametric sign test (Table 2). Comparing knees at a given degree of subluxation, the median distance measured increased for all degrees of subluxation, but the increase did not reach statistically significant (Sign test, Table 3). The intraclass coefficient for two observers is given in Table 4.

## DISCUSSION

In this study, we were looking for changes in distance between the posterior tibial cortex at the level of a theoretical tibial cut plane and the popliteal artery with flexion and tibiofemoral subluxation. We found significant increases in this distance with both increasing flexion of the knee and increased tibiofemoral subluxation.

Popliteal artery laceration is a rare, but devastating complication of total knee replacement. In a large meta‐analysis, Sundram et al. [22] reported an incidence of 0.05% or 54 per 100,000 cases and amputation or permanent neurological complications occurred in 21% after major vascular injury. Similarly, Dua et al. [8] reviewed the National Inpatient Sample database including 1,297,369 cases and found an incidence of 0.003% of reported popliteal artery injuries. Other case series and multi‐centre studies reported incidence of 0.03%–0.17% [1, 4, 6, 18, 19, 24].

The popliteal artery is tethered proximally as it exits Hunter's canal, and it then transverses distally and laterally, inclining obliquely to the distal border of the popliteus muscle. Here, it is tethered again where it divides into the anterior and posterior tibial arteries. Angiographic studies have demonstrated that the middle geniculate artery is coiled in extension and progressively uncoils in flexion to allow the popliteal artery to move posteriorly in the fossa [2, 25].

Most studies of the anatomy of the popliteal artery at the level of the knee demonstrate an increasing distance from the posterior cortex of the tibia to the popliteal artery with increasing knee flexion. However, Zaidi et al. [27], in an ultrasound study, found that the popliteal artery was closer to the tibia in flexion in 12 out of 20 healthy volunteers. However, volunteers were imaged on their sides, negating the effect of gravity and the ultrasound may have compressed the popliteal fossa. Shetty et al. [20] using duplex ultrasound also concluded that the popliteal artery moved closer to the tibia with knee flexion in 24% of patients studied. Other investigators, using magnetic resonance technology, have concluded that the Popliteal artery moves away from the tibia with knee flexion [16, 21].

Traditional knee replacement techniques, such as the gap balancing technique advocated by Insall and Ranawat or the measured resection technique described by Hungerford and Krackow all advocate resection of the proximal tibia with the knee in flexion. Most surgeons performing these techniques perform the proximal tibia resection in some degree of subluxation both to improve exposure and protect the popliteal artery [9, 11, 12, 14].

More recently, minimally invasive techniques, such as the quad‐sparing technique, have advocated cutting the tibia first with the knee relatively extended [15, 23]. Other minimally invasive techniques do not require flexion or subluxation of the knee before cutting the tibia [3, 5, 10]. If this puts the popliteal artery closer to the posterior tibia, it increases the risk of arterial injury.

Our study confirms prior work that the popliteal artery moves away from the tibia with increased flexion. This change reached statistical significance at both 90° and 115° of knee flexion. In addition, it is the first to our knowledge to show that increased tibial subluxation also increased the distance to the popliteal artery and, thus, the margin of safety for proximal tibial resection. In fact, in several specimens, the theoretical resection plane did not intersect the course of the popliteal artery. This finding is clinically significant, in that it indicates the risk of popliteal artery laceration with tibial resection in TKA is minimized by performing the resection with the knee in flexion and the tibia subluxed.

The strength of our study is that it was performed with venography under laboratory conditions. The leg was held in an exact position of flexion which was confirmed with radiographic measurement. Forward subluxation and external rotation were likewise radiographically controlled. In traditional knee replacement techniques, the tibia is externally rotated in flexion to increase the separation between the popliteal artery and posterior tibia. Therefore, limited external rotation represents a worst‐case scenario. A posterior slope of 7° of the theoretical cut plane was selected because the increased slope brings the posterior cut plane closer to the trifurcation and is thus a worst‐case scenario. Seven degrees of slope is the highest tibial slope recommended in any common TKA system [28]. Excellent visualization of the popliteal artery with radiocontrast was achieved. Precise measurements were accomplished with the use of a magnification control marker and a fixed reference point for the posterior tibia. This allowed for a highly reproducible technique and yielded very consistent findings with small standard deviations in the measurements. The measurements were very consistent between observers, except in the case of non‐intersections (infinity measures). Some measurements were made in an area where the popliteal artery is strongly sloping away from the extended simulated cut line on the tibia, therefore even a tiny difference in measurement technique between observers could result in a non‐intersection between the line and the artery. We believe that these results are useful in understanding the risks of popliteal artery injury in total knee replacement.

A limitation of the study is the small sample size. Infinity measures (non‐intersection) also presented a problem in the data analysis. An alternative test could be the Wilcoxon Sign Rank test which takes into account the magnitude, as well as the direction of the ranks of measures.

## CONCLUSION

The distance between the posterior cortex at the level of tibial resection for TKA and the popliteal artery increases with both flexion of the knee and subluxation of the tibia. This should be taken into consideration to minimize the risk of popliteal artery injury, especially when performing certain types of minimally invasive approaches.

## AUTHOR CONTRIBUTIONS

Study origin, design and data analysis were performed by first, second and fourth authors. The manuscript was written by the first author, and all authors read, edited and approved the final manuscript. The third author performed the literature search, drafted and edited the manuscript, and was involved in the final submission.

## CONFLICT OF INTEREST STATEMENT

The authors declare no conflict of interest.

## ETHICS STATEMENT

This study did not involve humans, human data, or animals and therefore did not require approval from the ethical committee.

## Data Availability

Data are available based on reasonable request from the corresponding author.
